# Depth Cues and Perceived Audiovisual Synchrony of Biological Motion

**DOI:** 10.1371/journal.pone.0080096

**Published:** 2013-11-14

**Authors:** Carlos César Silva, Catarina Mendonça, Sandra Mouta, Rosa Silva, José Creissac Campos, Jorge Santos

**Affiliations:** 1 Department of Informatics, University of Minho, Braga, Braga, Portugal; 2 Department of Psychology, School of Medicine and Health Sciences, Carl von Ossietzky University, Oldenburg, Lower Saxony, Germany; 3 Department de Psicologia Bàsica, University de Barcelona, Barcelona, Catalonia, Spain; 4 INESC Porto, Instituto de Engenharia de Sistemas e Computadores, Porto, Porto, Portugal; 5 Department of Basic Psychology, School of Psychology, University of Minho, Braga, Braga, Portugal; 6 Department of Informatics, School of Engineering, University of Minho, Braga, Braga, Portugal; 7 HASLab (High-Assurance Software Laboratory), INESC TEC Porto, Porto, Portugal; 8 Centro Algoritmi, University of Minho, Guimarães, Braga, Portugal; 9 Centro de Computação Gráfica, Guimarães, Braga, Portugal; University of Muenster, Germany

## Abstract

**Background:**

Due to their different propagation times, visual and auditory signals from external events arrive at the human sensory receptors with a disparate delay. This delay consistently varies with distance, but, despite such variability, most events are perceived as synchronic. There is, however, contradictory data and claims regarding the existence of compensatory mechanisms for distance in simultaneity judgments.

**Principal Findings:**

In this paper we have used familiar audiovisual events – a visual walker and footstep sounds – and manipulated the number of depth cues. In a simultaneity judgment task we presented a large range of stimulus onset asynchronies corresponding to distances of up to 35 meters. We found an effect of distance over the simultaneity estimates, with greater distances requiring larger stimulus onset asynchronies, and vision always leading. This effect was stronger when both visual and auditory cues were present but was interestingly not found when depth cues were impoverished.

**Significance:**

These findings reveal that there should be an internal mechanism to compensate for audiovisual delays, which critically depends on the depth information available.

## Introduction

 To perceive synchrony in audiovisual events we face intricate problems because of the relative timing of visual and auditory inputs. These problems are related both to physical and neural differences underlying sound and light propagation and processing. When a natural audiovisual event occurs, the visual and auditory signals are synchronic at the origin, since they are caused by the same physical source and thus emitted at the same time. However, there are significant differences in the propagation time for light and sound, as sound takes about 3 milliseconds (ms) to travel 1 meter (m) and light travels approximately 299 792 m in 1 ms. Nevertheless, in our daily life audiovisual stimuli are still perceived as synchronic at the source. Given the abovementioned sound delay of about 3ms per meter of distance from the visualized object, it is not obvious how audiovisual stimuli can so often be perceived as a unitary phenomenon.

 In fact, common range distances from stimuli sources to observers are often large enough to create a considerable gap between the arrival times of visual and auditory signals. Furthermore, differences at the level of transduction times were also found [2,3], with sound being transduced faster (~ 1 ms; see 1) than light (~50 ms). However, as these neural temporal differences are fairly constant, observers become adapted to this difference due to a long history of exposure to such “veridical” neural lags [4]. Nevertheless, it remains to be explained how we perceive an audiovisual event as unitary when the audio and the visual streams physically arrive to the observer at variable asynchronies. 

 Several studies have shown that audiovisual integration does not require temporal alignment between the visual and the auditory stimuli. We still perceive as synchronic visual and auditory stimuli that are not received or emitted at the same time (e.g. [5-11]). Notwithstanding, temporally mismatched stimuli can only be perceived as synchronic when keeping the onset difference between sound and image within certain limits. These limits have been termed window of temporal integration (WTI) [12,13]. In multisensory perception this phenomenon can be defined as the range of temporal differences on the onset of two or more stimuli of different modalities where these are best perceived as a unitary multisensory stimulus. As Vroomen and Keetels [12] pointed out, the main reason why despite these differences signals from different sensory modalities are perceived as being synchronic is because the brain judges as synchronic two stimulation streams that arrive within a certain amount of temporal disparity.

 Research on this phenomenon has provided us with surprising findings. A large number of studies on audiovisual temporal alignment have found that we perceive stimuli from different modalities as being in maximal synchrony if the visual stimulus arrives at the observer shortly before the auditory stimulus (e. g., [5,8,9,14]). This finding has been termed the *vision-first bias* [13,12]. In a work that boosted the scientific discussion on the vision-first bias [9], Sugita and Suzuki used a temporal-order judgment (TOJ) task to assess the perceived temporal relation between the emission of a sound (a burst of white noise) and a brief light flash. The flashes of light were displayed from LEDs located at distances of 1, 5, 10, 20, 30, 40 and 50 m. The sound was always transmitted by headphones but was compared with the visual stimuli at different distances. In their paper, Sugita and Suzuki reported that the stimulus onset asynchrony (SOA) that provides the best perception of synchrony is always a positive one (i.e., sound lagging) and most importantly, when the distance of visual stimuli increases, larger lags are observed at the point of subjective simultaneity (PSS). This positive correlation can be described by an increment of about 3 ms in the PSS for each one-meter increment in the visual stimulus distance. These results are roughly consistent with the velocity of sound, at least up to 20 m of visual stimulus distance, and can be quite well predicted by a linear model based on this physical rule. Thus, it seems that the brain takes sound propagation into account when judging synchrony, relying on distance information to *compensate* for the differences on the stimuli’s timing. Other studies have also pointed to a perceptual mechanism of compensation for differences in propagation velocity (e.g., [5,8,15]), further suggesting that we *resynchronize* the signals of an audiovisual event by shifting our PSS in the direction of the expected audio lag. 

 Nonetheless, while WTIs have been widely employed to explain the perception of audiovisual synchrony, some researchers have been reluctant to accept a perceptual mechanism that compensates for sound-transmission delays [16,17,18]. For them, a mechanism like this would be a remarkable computational feat, because it would require an implicit knowledge of physical laws, namely the knowledge of sound propagation velocity. Moreover, some studies have failed to find evidence for such a compensation mechanism [17,18]. Lewald and Guski [17] tried to replicate the findings of Sugita and Suzuki [9] in a less artificial setting. Using the same kind of stimuli (sound bursts and LED flashes) but co-located, they found no distance compensation. In fact, the PSS shifted in the opposite direction. In this experiment participants had the best perception of synchrony when auditory and visual signals were synchronic in their arrival at the observer’s sensorial receptors. As Lewald and Guski pointed out, “this conclusion is in diametral opposition to the study of Sugita and Suzuki” (p. 121), and this discrepancy might be due to problematic procedures used in the former study. According to the authors, there are two main problems in Sugita and Suzuki’s study, both related to the stimuli. Firstly, the sound stimuli were not *co-located* with the visual stimuli and consequently there was no auditory distance information. Secondly, the luminance of the visual stimuli was increased to compensate for the light intensity attenuation with distance. However, by doing this, they kept the perceived stimuli’s luminance constant, thus providing a cue that is incongruent with the expected information on distance increment. Moreover, parameters such as size and contrast, quite relevant in the perception of distance, were not kept constant and could have been affected by the luminance manipulation.

 According to Lewald and Guski [17], those manipulations made the experimental design inconsistent with everyday life. Arnold and colleagues [18] reached similar results and also highlighted the same problems in Sugita and Suzuki’s (2003) work. However, both studies of Lewald and Guski [17] and of Arnold and colleagues [18] still present potential problems in the simulation of distance, namely:

1. By conducting the experiment in open-field, Lewald and Guski [17] failed to provide the optimal conditions for auditory distance information 1 of the most powerful auditory depth cues is the ratio of energies of direct and reflected sounds [19], which is almost absent in free-field stimulation. Also, the most important depth cue in a situation of open-field – loudness – is frequently and erroneously perceived as the level of the sound itself in the absence of relative loudness cues, which can cause misjudgments of stimuli distance;
2. The use of artificial stimuli, such as flashes and beeps, eliminated two other relevant depth cues: familiar size of the visual stimuli and familiar loudness of the auditory stimuli.
3. Arnold and colleagues [18] manipulated the angular size and velocity (i.e. retinal size and velocity) of their visual stimuli to ensure that the size and velocity of the stimuli appeared constant while distance increased. This poses the same problem of incongruent depth cues pointed out by Lewald and Guski [17] to the work of Sugita and Suzuki [9].

Considering the above controversy and taking into account the main critiques regarding previous studies on compensatory mechanisms, here we report findings that offer a clearer answer to the question: How can we perceive an audiovisual event as such, if the audio and the visual signals physically arrive at different times to the observer? 

 To do so, we used audiovisual biological motion stimuli. Recent findings show that in the study of audiovisual synchrony [6,11] biological motion stimuli are preferable over rigid motion stimuli. The use of point-light displays with biological motion has been previously pointed out as an important factor in simultaneity judgment tasks. In a study where participants had established a baseline PSS to an audiovisual stimulus consisting of footage of a professional drummer playing a conga drum, Arrighi and collaborators [6] found that the PSS was not different when the visual stimulus was a computerized abstraction of the drummer with the same movement (biological) presented in the footage. However, when the same artificial visual stimulus had an artificial motion pattern (constant velocity) the PSS was significantly different, even when the frequency was the same in both conditions. Petrini, Holt, and Polick [20] have found that a non-natural orientation of a point-light drummer can affect the simultaneity judgment of non-musical expert participants, thus showing that naturalistic representations are preferred in this kind of tasks. 

 Biological stimuli also allow for high levels of control and manipulation of critical parameters for visual and sound depth perception. Namely, angular and familiar size, angular velocity, elevation, intensity, contrast, and perspective for visual distance judgments; and sound pressure level, high frequency attenuation, and reflected sound for auditory distance judgments. Thus, in order to assess the existence of a mechanism that compensates for the differences in propagation velocity we presented the audiovisual biological stimulus at several distances in a controlled environment simulating the real physical world. Additionally, by manipulating the number of depth cues presented in the stimuli we assessed their relevance in the compensation mechanism. 

 In short, we expect to support the argument of perceptual compensation if we find a positive relation, close to the rule of physical propagation of sound, between the shift of the PSS (towards an audio lag) and the distance of the stimuli. We also expect this relation to be dependent on the number and quality of the depth cues. The results from this experiment will be discussed in light of their implications for the conceptualization of the mechanisms of simultaneity constancy. 

## Method

### Ethic Statement

 The experiment was approved by the Direction Board of the Doctoral Program in Informatics, Department of Informatics, University of Minho. All participants gave their written informed consent. The experiment was conducted in accordance with the principles stated in the 1964 Declaration of Helsinki.

### Participants

 Four participants, aged 21-33 years old, underwent visual and auditory standard screening tests and had normal hearing and normal, or corrected to normal, vision. All were voluntary university students or researchers and all gave written informed consent to participate in the study. Two had some background knowledge about the thematic of the study and the remaining two were naïve as to the purpose of the experiment. 

### Stimuli and Materials

 The experimental tasks were performed in a darkened room at the Laboratory of Visualization and Perception in the University of Minho. For the stimuli presentation we used a cluster of PCs with NVDIA^®^ Quadro FX 4500 graphic boards with custom software running on top of OpenGL and VR/Net Juggler. A 3chip DLP projector Christie Mirage S+4K with a resolution of 1400x1050 pixels and a frame rate of 60Hz was used for the projection of the visual stimuli. The area of projection was 2.80 m high and 2.10 m wide. The sound was presented by a computer with a Realtec Intel 8280 IBA sound card through a set of Etymotics ER-4B in-ear phones. Latencies between visual and auditory channels were measured and adjusted using a custom-built latency analyzer consisting of an Arm7 microprocessor coupled with light and sound sensors.

 Three experimental conditions differing from each other in the number of depth cues were presented. The “audiovisual depth cues” condition presented visual and auditory depth cues coherent with the simulated presentation distance. In the “visual depth cues” condition only the visual depth cues varied coherently with the simulated presentation distance. The “reduced depth cues” condition presented both the visual and auditory depth cues impoverished. 

#### Auditory Stimuli

In the “audiovisual depth cues” condition the auditory stimulus consisted in a binaural sound recorded using a Brüel&Kjaer^®^ head and torso simulator Type 412SC. An anechoic step sound was emitted trough a Brüel&Kjaer^®^ omnidirectional Loudspeaker Type 4295 inside a sports pavilion 24 m wide, 50 m long and 11 m high. The step sound was recorded at distances of 10, 15, 20, 25, 30, and 35 m from the head and torso simulator, located 6 m from one end of the sports pavilion (see Sound S1). The anechoic step sound came from the database of controlled recordings from the College of Charlston [21] and corresponds to the sound of a male walking over a wooden floor and taking one step. In the two remaining conditions the auditory stimulus was an auralized sound of the above-referred anechoic recording, with directional cues matching the visual stimulus, but in free field and without air attenuation with distance. 

#### Visual Stimuli

**Figure 1:**
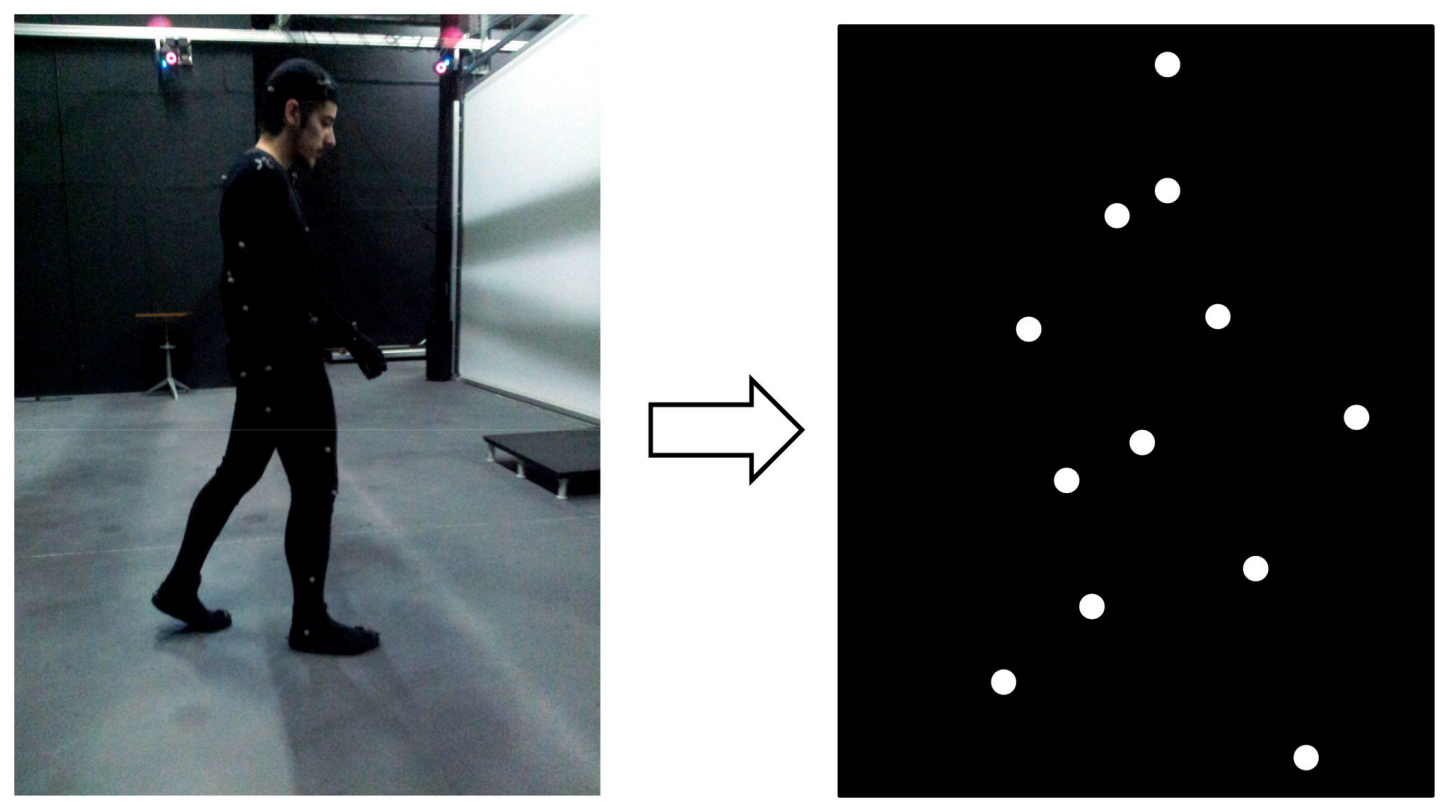
Point Light Walkers Capture. On the left panel a frame of a participant in a session of biological motion capture is presented. The markers placed on the suit in specific locations, are designed to reflect near infrared light transmitted by the 6 cameras. This way we could record in real time the position (along 3 axes) of each marker in order to design an animated representation of the human body movement (image on the right panel). The subject of the photograph "has given written informed consent, as outlined in the PLOS consent form, to publication of their photograph.

Different visual stimuli according to the experimental condition were presented to participants. In both the “audiovisual depth cues” and the “visual depth cues” conditions the visual stimuli were Point-Light Walkers (PLW) moving in a front-parallel plane to the observer and taking one step aligned with the center of projection (see supporting information). The PLW was walking inside a simulated room with the same dimensions of the sports pavilion referred before. The PLW was composed of 13 white dots (54 cd/m^2^) that moved against a black background (0.4 cd/m^2^) and was generated in the Laboratory of Visualization and Perception (University of Minho) from motion data captured using a Vicon^®^ system with 6 MX F20 cameras and a set of custom LabVIEW routines. All stimuli corresponded to the correct motion coordinates of a 1.87 m high, 17 year-old male, walking at a velocity of 1.1 m/s. The duration of the visual stimulus was variable from scene to scene in order to avoid the use of a fixed time between the beginning of the scene’s projection and the occurrence of the step. Thus, there were three different durations: 1.08 ms, 1.12 ms and 1.17 ms; and the PLW step occurred at the 527^th^ ms in the minimum duration; at the 547^th^ ms in the medium duration; and at the 569^th^ ms in the maximum duration (Figure 1). 

To simulate the six stimuli distances (10, 15, 20, 25, 30 and 35 m), the visual angular size and angular velocity of the stimuli was changed according to the expected changes in the physical world.

Using PLWs, two important pictorial depth cues were made available: *familiar size* and *elevation*. Furthermore, these visual stimuli also presented two dynamic depth cues: the *amplitude of the step* (wider steps represent a closer presentation) and the *angular velocity* (a smaller angular velocity translates into a farther distance of presentation). The PLW allowed the coordination of these depth cues by decreasing the angular size and velocity, as well as by decreasing the angular size of the dots composing it, and by gradually increasing its elevation according to the stimuli distance.

**Figure 2:**
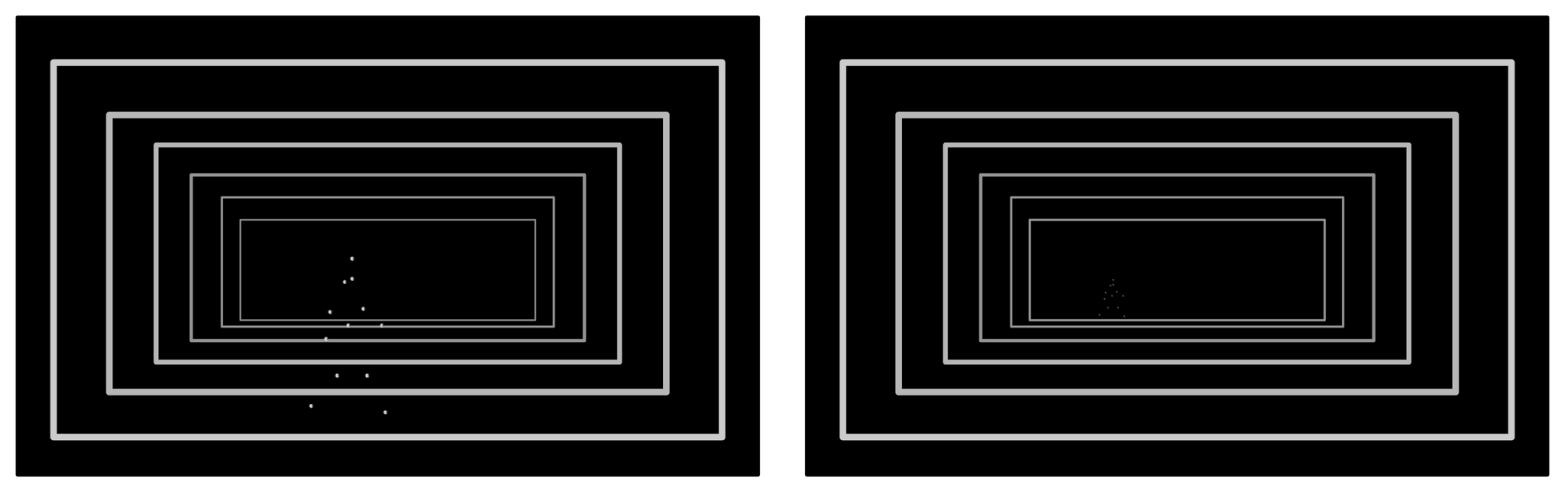
Perspective Depth Cue. See Video S1.

The room simulation was made using perspective depth frames. The floor and wall lines were virtually located at 10, 15, 20, 25, 30, and 35 m from the observer. Thus, the PLW corresponding to each one of these distances was presented as walking on top of the correspondent ground line (Figure 2).

In the “reduced depth cues” condition, most depth cues were eliminated from the visual stimuli. These stimuli presented no room perspective cues since only the feet were presented, with a random size of dots and at a constant elevation. This way we also eliminated the bodily cues of familiar size. Thus, the single depth cue available was the amplitude of the step, with wider steps meaning a closer presentation.

#### Visual and Auditory Stimuli Relation

**Figure 3:**
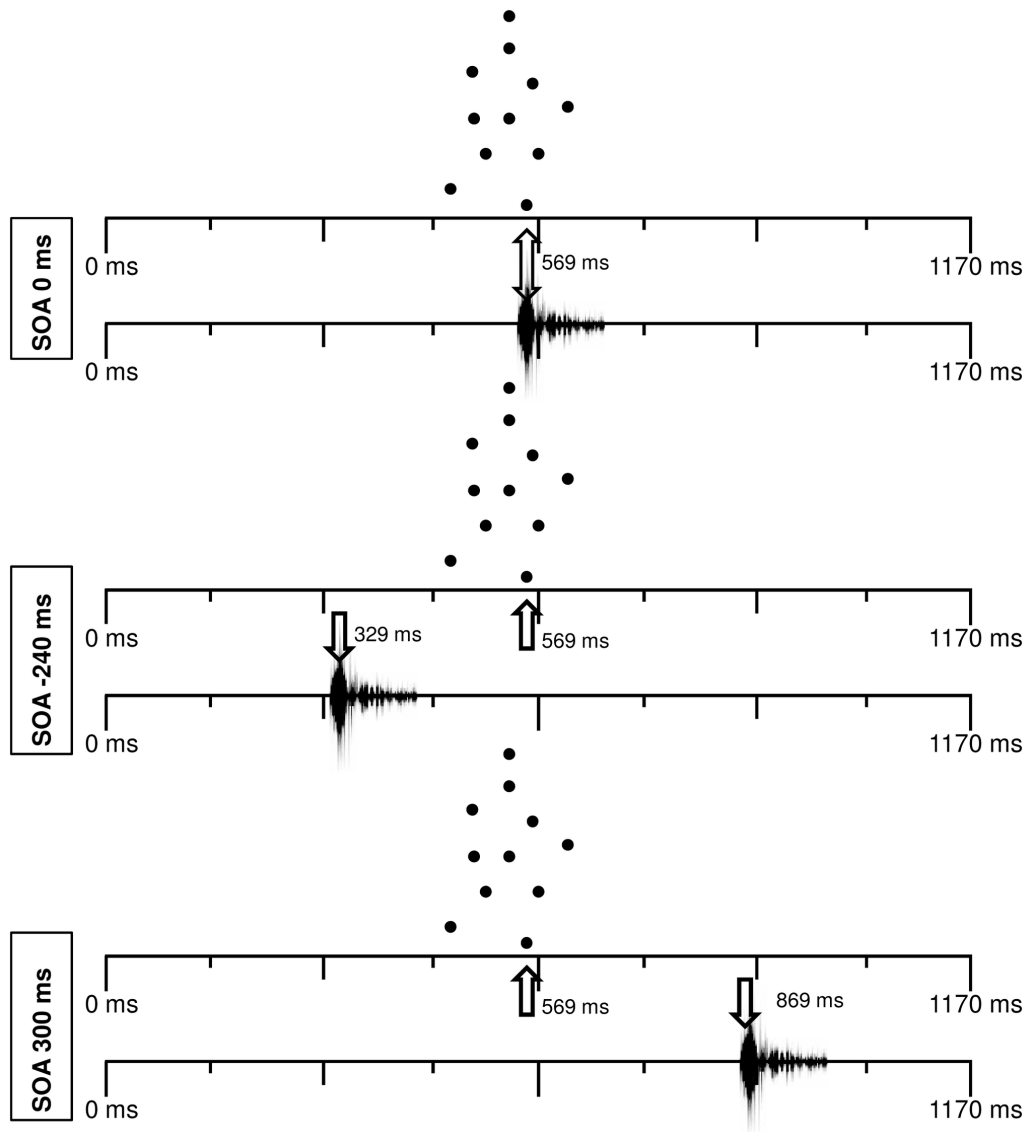
Examples of audiovisual stimuli where we can see when steps occur. We can see the temporal relations between the occurrence of the visual step and the occurrence of the auditory step in the SOAs 0 ms, -300 ms, and +300 ms.

In order to present several audiovisual events, the visual and the auditory stimuli were combined in 19 different stimuli onset asynchronies (SOAs) for several distances (Figure 3). The SOAs took the following values:

. For a PLW at a distance of 10, 20, or 30 m: -240 ms; -210 ms; -180 ms; -150 ms; -120 ms; -90 ms; -60 ms; -30 ms; 0 ms; 30 ms; 60 ms; 90 ms; 120 ms; 150 ms; 180 ms; 210 ms; 240 ms; 270 ms; 300 ms;

. For a PLW at a distance of 15, 25, or 35 m: -225 ms; -105 ms; -165 ms; -135ms; -105 ms; -75 ms; -45 ms; -15 ms; 0ms; 15 ms; 45 ms; 75 ms; 105 ms; 135 ms; 165 ms; 195 ms; 225 ms; 255 ms; 285 ms. 

Negative values indicate that sound was presented first, and positive values that sound was presented after the visual stimulus. The reason why we had a different spectrum of SOAs for even and odd distances was twofold: Firstly, we wanted to always present the theoretical values that, according to the compensation for sound velocity hypothesis, would provide the best sensation of audiovisual synchrony (for e.g., 30 ms for stimuli at 10 m from the observer, 45 ms at 15 m, and so on). Secondly, we wanted to keep the experimental sessions within a reasonable duration to avoid fatigue effects. Therefore, we had two groups of 19 SOAs presented, each one at 3 distances comprising a total of 114 different audiovisual stimuli.

### Procedure

 In each experimental session we presented the PLW at 3 different distances in a random order. All stimuli were randomly presented with 40 repetitions and with a given duration according to the condition: 5.5 s in the “audiovisual depth condition” and 2.5 s in the “visual depth condition” and in the “reduced depth condition”. There was an inter-stimuli interval of 1.5 s for all conditions. 

 Before each experimental session the participants were shown 10 repetitions of an audiovisual stimulus in which the sound appeared with a 300 ms lead, and 10 other repetitions of an audiovisual stimulus in which the sound appeared with a 330 ms lag. This preliminary session was taken in order to check if participants were able to perceive any kind of asynchrony. None of the SOAs used in this preliminary session were then used in the experimental session. 

 At the beginning of the experimental session the following instructions were given: “You are going to participate in a study in which you will be presented with several audiovisual scenes of a PLW walking at a certain distance. I want you to pay close attention to the audiovisual scene, because you will have to judge its audiovisual synchrony. In this scene you will see a walker taking one step and you will hear his step sound. The distance of presentation may vary between 10, 20, and 30 m (or 15, 25, and 35 m, in some trials). After each scene, if you think that the auditory and the visual streams were synchronized click the right button; otherwise, if you think that the auditory and the visual streams were not synchronized click the left button. Please give your answer only after the visual and auditory stimuli presentation”. 

 The participant was seated in a chair 4 m from the screen and in line with the center of the projection area. In each scene the participant was presented with a PLW walking from left to right and taking one step at a velocity of 1.1 m/s, while listening trough in-ear phones to one step in a given temporal relation with the visual stimulus. After the presentation of each audiovisual stimulus and during the inter-stimulus interval, the participant had to answer in a two-key mouse according to the instructions. 

 The experimental sessions were blocked by condition and the conditions’ order was randomized between participants.

## Results

 The individual analyses of PSS and WTI for all participants are shown in Table 1. PSSs were obtained by adjusting a Gaussian function to the data and WTIs were calculated following the method presented in the review of Vroomen and Keetels [12].

**Table 1:** Individual values of the PSS and WTI.

**Part.**	**Condition**	**PSS 10m (WTI)**	**PSS 15m (WTI)**	**PSS 20m (WTI)**	**PSS 25m (WTI)**	**PSS 30m (WTI)**	**PSS 35m (WTI)**	**Linear Fitting**	**Adjust**.( R^2^)
1*	“Audiovisual Depth Cues”	16 (77)	2 (73)	18 (80)	26 (88)	60 (100)	57 (84)	y = 2.21x - 19.9	0.71
	“Visual Depth Cues”	54 (98.5)	69 (113)	69 (106)	80 (96.5)	113 (78)	95 (86.3)	y = 1.96x + 35.62	0.71
	“Reduced Depth Cues”	89 (108)	83 (117)	86 (98.5)	62 (88)	75 (86.5)	61 (79.5)	y = -1.07x + 100.3	0.60
**2**	“Audiovisual Depth Cues”	-10.5 (102)	-13 (89)	30 (104)	36 (138)	74 (100)	91 (106)	y = 4.42x - 64.9	0.93
	“Visual Depth Cues”	35 (86)	43 (70)	47 (77)	52 (69.5)	43 (87)	71 (60.5)	y = 1.04x + 24.88	0.54
	“Reduced Depth Cues”	46 (66)	31 (69)	40 (60.5)	27 (62)	10 (49.5)	18 (68)	y = -1.20x + 55.50	0.66
3*	“Audiovisual Depth Cues”	22 (126)	-3 (118)	28 (114)	20 (117)	75 (105)	84 (125)	y = 3.06x - 31	0.62
	“Visual Depth Cues”	41 (107)	40 (109)	45 (104)	47 (116.5)	59 (90.5)	63 (116.5)	y = 0.97x + 27.16	0.85
	“Reduced Depth Cues”	2 (116)	-11 (115)	-4 (113.5)	-13 (121)	-18 (109)	5 (117)	y = -0.07x - 4.88	0.42
**4**	“Audiovisual Depth Cues”	50 (253)	69 (232)	68 (161)	76 (213)	106.5 (124)	126 (160)	y = 2.86x + 18.23	0.88
	“Visual Depth Cues”	103 (132.5)	88 (106)	117 (125)	108 (119)	96 (101)	100 (100.5)	y = 102	0.71
	“Reduced Depth Cues”	-12 (91.5)	-13 (103)	-25 (91)	-30 (64.5)	-19 (101.5)	-13 (91)	y = -0.17x - 14.73	0.71

The values are presented in ms and for the several distances in each stimulus condition. In the last column the equations and the values of adjustment for each of the linear functions fitted to the individual data are presented. The asterisks signal the participants that had some background knowledge about the thematic of the study.

 Two participants had some background knowledge about the thematic of the study and the remaining two were naïve to the purpose of the experiment. According to independent sample t-tests there was no significant difference between both groups with regard to the PSS values in the “reduced depth cues” condition (t (22) = 1.99, *n.s.*), in the “visual depth cues” condition (t (22) = -0.98, *n.s.*) and in the “audiovisual depth cues” condition (t (22) = -1.68, *n.s.*). Regarding the WTI values, there were no significant differences among participants regarding their background knowledge about the study’s thematic in the “visual depth cues” condition (t (22) = .962, *n.s.*). There were, however, significant differences regarding the WTI values in the “reduced depth cues” condition (t (22) = 4.4, *p<.01*) and in the “audiovisual depth cues” condition (t (22) = -2.78, *p<.05*). Participants with background knowledge had higher values of WTI in the “reduced depth cues” condition, but lower values of WTI in the “audiovisual depth cues” condition.

**Figure 4:**
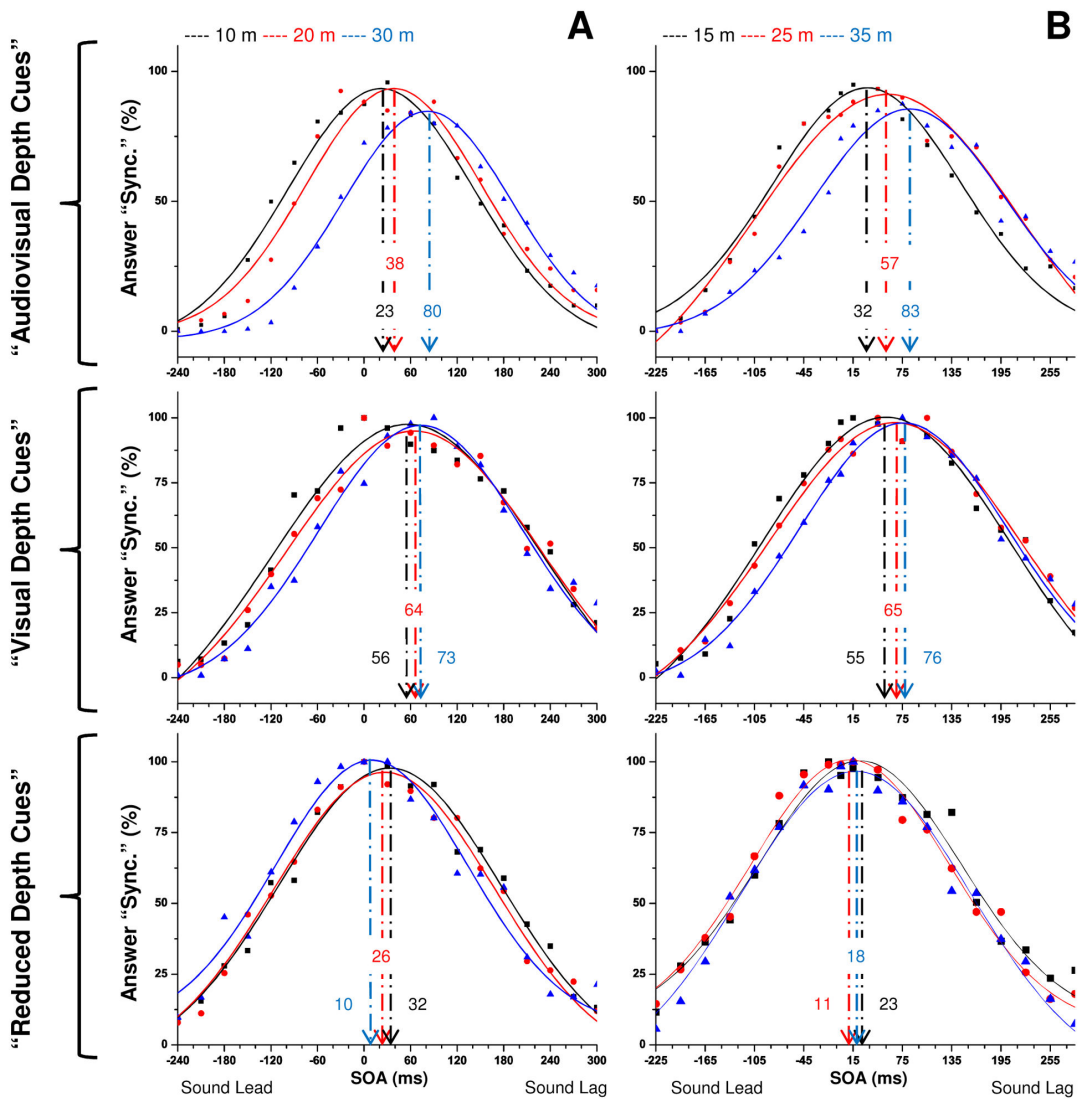
Proportion of “synchronized” answers as a function of the SOA for a data pool of distances of 10, 15, 20, 25, 30 and 35 m in the three experimental conditions. Each proportion was calculated using a pooled data of 160 answers from the 4 participants. Panel A shows the answer distributions for distances of 10, 20, and 30 meters; panel B shows the answer distributions for distances of 15, 25, and 35 meters. A fit of a Gaussian function was performed in order to get the PSS and WTI values for each distance of stimulation. An arrow indicates the PSS for each stimulus distance.

Since differences between both groups were not found, a One-way analysis of variance (ANOVA) was calculated for all participants' PSS values. This revealed differences between the participants’ PSS values for the “reduced depth cues” condition (F (3,20) = 93.16, *p<.01*), the “visual depth cues” condition (F (3,20) = 20.43, *p<.01*) and the “audiovisual depth cues” condition (F(3,20) = 3.32, *p<.05*). Nevertheless, the Scheffé Post-hoc tests show that these differences between participants are significant in all the comparisons of the “reduced depth cues” condition (with the exception being the comparison between participant 3 and participant 4) and in some comparisons of the “visual depth cues” condition, where participant 1 has a significantly higher PSS than participant 2 (p<.01), and participant 4 has a PSS significantly higher than participant 2 (p<.01) and participant 3 (p<.01). The individual analyses of PSS and WTI for all participants show similar response patterns across participants (i.e., the slope of the linear fitting to the PSS values increases when we go from conditions with less depth cues to conditions with more depth cues). Therefore, we chose to pool all the individual data for a more detailed analysis. Figure 4 shows the fitting of a Gaussian function to the data pool for distances of 10, 20, 30 m (graph on the left) and 15, 25, 35 m (graph on the right), for each one of the conditions. All the data, grouped by distance, conformed well to the Gaussian fittings. 

 In both the “audiovisual depth cues” and “visual depth cues” conditions, the peak of the Gaussian curve progressively moves towards a higher sound delay as the stimulus distance increases. This increment is generally lower in the “visual depth cues” condition, where there is a difference of only 20 ms between the lowest (in the 15 m presentation) and the highest (in the 35 m presentation) PSS, while in the pooled data of the “audiovisual depth cues” condition this difference is of 60 ms (with the lowest PSS at the 10 m presentation and the highest at 35 m). However, in the “reduced depth cues” condition the peak of the Gaussian curve hardly moves from one distance to another (especially in the odd group of distances) and when it moves, it does so in the direction of a lower sound delay. This was the only condition where several PSSs with a value close to zero (see distances 25 and 30 m) were found. A One-way ANOVA shows significant differences between conditions regarding the PSS (F (2, 15) = 11.9, *p < .01*), and the Scheffé Post-hoc tests revealed that these differences are significant when we compare the PSSs in the “reduced depth cues” condition with the PSSs in the “visual depth cues” condition (p < .01) and with the PSSs in the “audiovisual depth cues” condition (p < .05).

**Figure 5:**
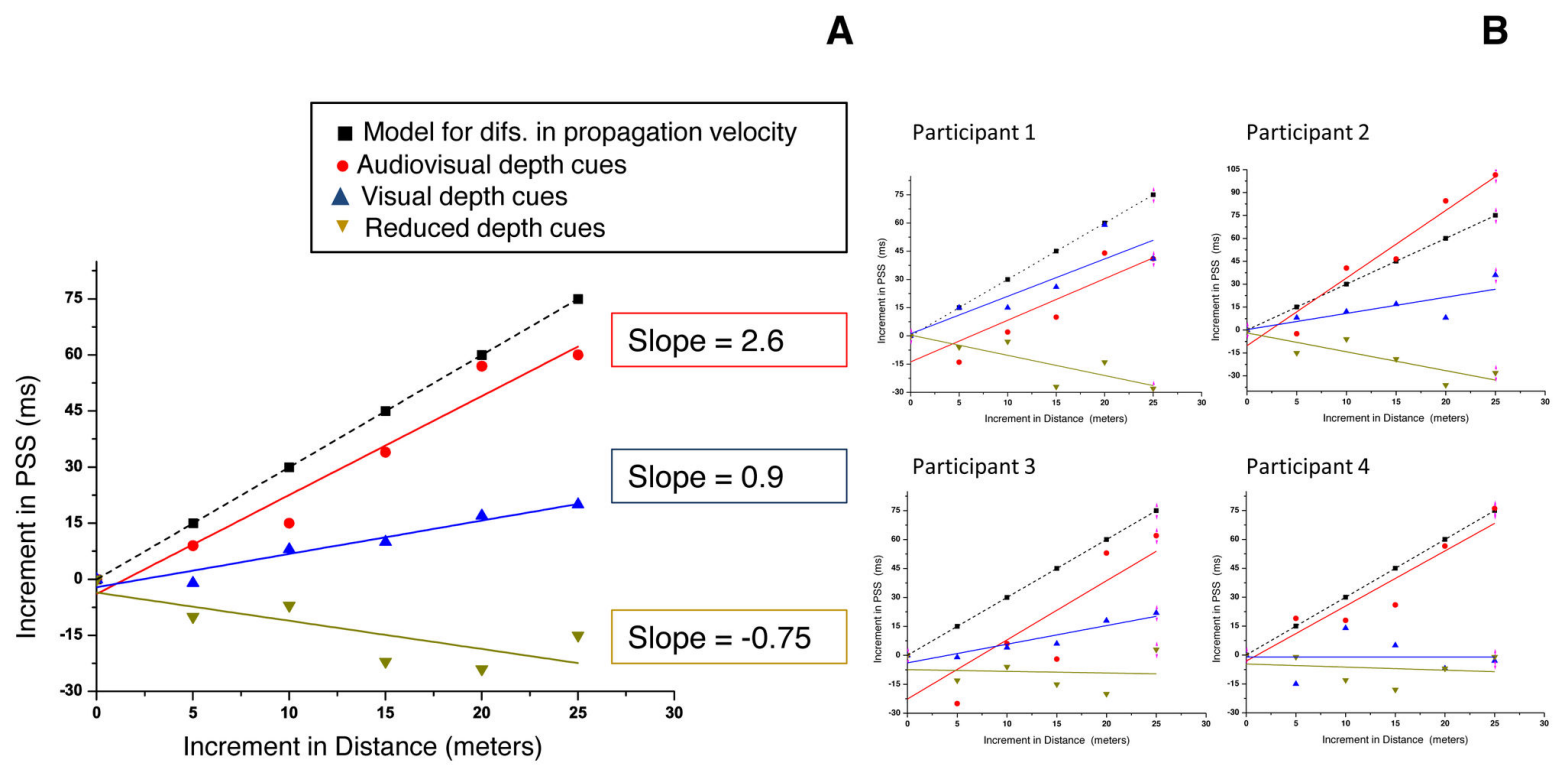
Increment in the PSS as a function of increment in the stimulus distance, regarding the first distance of presentation. Black squares correspond to the theoretical values predicted by a mechanism that compensates for differences in propagation velocity. Red dots are the PSS found for each distance in the “AV depth cues” condition. Blue triangles are the PSS found for each distance in the “visual depth cues” condition and orange triangles are the PSS found for each distance in the “reduced depth cues” condition. A fit of a linear function was performed in each group of data. Panel A shows the graph for the pooled data, and Panel B show the same graph for the individual data.

Figure 5 plots the increment in the PSS as a function of the increment of the stimulus distance regarding the first distance of presentation for each condition. Here we can compare the way the PSS changes across conditions with a model for internal compensation of the slower propagation velocity of sound. A linear function was fitted to the PSSs obtained in the three conditions. A good adjustment (r^2^ = .94, F (1,4) = 84.8, *p < .01*) on the fitting of the function y = 2.6x to the results in the “audiovisual depth cues” condition was obtained. In the “visual depth” condition the best linear function was y = 0.9x, with a good fit (r^2^ = .93; F (1,4) = 63.84, *p < .01*). Similarly, a linear function was fitted to the PSSs obtained in the “reduced depth cues” condition (y = -.75x), but only with a rough adjustment (r^2^ = .5, F (1,4) = 5.77, *p < .1*).

Figures 4 and 5 clearly show that the conditions “audiovisual depth cues” and “visual depth cues” present different tendencies when compared with those from the condition “reduced depth cues”. While the PSS from the “audiovisual depth cues” and “visual depth cues” condition increases with distance, the PSS from the “reduced depth cues” condition seems to decrease with distance. In fact, correlation tests show that the PSS in the “audiovisual depth cues” condition is positively and significantly correlated with distance (r_sp_ = 1, *p < .001*) and the same is true for the PSS in the “visual depth cues” condition (r_sp_ = .943, *p < .01*). On the other hand, PSS in the “reduced depth cues” condition is just marginally correlated with distance (r_sp_ = -.77, *p < .1*), and in the opposite sense: higher PSSs are associated with lower distances.

## Discussion

 Our study aimed to address the long-standing debate on the existence of an internal mechanism to compensate for delays of signal propagation. To do so, we presented audiovisual biological stimuli at several distances and with a wide range of stimulus onset asynchronies. Crucially for this study, we manipulated the amount of distance cues in three experimental conditions: audiovisual depth cues, visual depth cues and reduced depth cues.

 Results from the first two experimental conditions revealed a systematic shift of the point of subjective simultaneity in the direction of greater audio lags with greater distance. Therefore, results support the existence of an internal compensation mechanism for varying stimuli delays with distance. Interestingly, compensatory evidences were not found in the “reduced depth cues” condition. Indeed, that condition was so impoverished that only visual angular velocity – a relatively poor distance cue – was available. Therefore, the internal compensation mechanism may depend on the amount and quality of depth cues available. This interpretation is further supported by the fact that in both conditions where evidence for such mechanism was found, the steepness of the function was not the same. In the “audiovisual depth cues” condition there was a steeper function, closer to that expected from the actual physical delays; in the “visual depth cues condition”, this function was smoother. We conclude that the proposed compensation mechanism might not work in an all-or-nothing way, but there might exist intermediate levels of compensation.

 We should call attention to a phenomenon that might have enhanced the increment in the PSS value in the “audiovisual depth cues condition”. In this condition, the mean of the Gaussian curves adjusted to the results in the 30 and 35 meters presentations never reached a value of 100% of synchrony responses. This means that the synchrony judgment was harder at farther distances, increasing the uncertainty of this type of judgment. The angular size of the dots representing the feet is relatively smaller at farther distances, which adds difficulty for the participant to know the moment of synchrony, as judgment becomes a more visually demanding task. This problem is not present in the “reduced depth cues” condition since we randomized the angular size of the dots representing the feet. Thus, we have to note that increasing the difficulty of the task, together with a vision-first bias can contribute for higher values of PSS. 

 From our findings, it stands out that depth cues might affect results found in synchrony judgments. However, at this stage we are not able to provide an exact account on what is guiding a compensatory pattern of response in some conditions. It could be argued that such a mechanism emerged due to the enhanced realism of the stimuli, or due to the causality between visual and auditory stimuli. In any case, we can assume that both factors contributed to a greater perceived unity between the multisensory signals. Future studies should focus on the relative effects and weights of different cues, different stimuli, and different settings and, with this in mind, a critical review of previous studies in this field might reveal that data obtained so far was not necessarily contradictory, but mostly the result of different experimental setups. 

 Nonetheless, our experiment still presents one methodological issue that has been considered a limitation in other studies: audiovisual stimuli might not have been perceived as co-localized, or could only be “imagined” as such. In fact, having found no evidence for perceptual compensation, Arnold and collaborators [18], Lewald & Guski [17], and Heron and collaborators [22] pointed out this feature as a partial factor for the evidence of compensation in other works. Indeed, in the present study, stimuli were not co-localized in the “visual depth cues” or in the “reduced depth cues” conditions. In the “audiovisual depth cues” condition stimuli were theoretically co-localized, due to a complete virtualization of both visual and auditory signals in space. In that condition, the in-ear phones were the only means of projection. Subjects should hear the sound as if emitted from an external source, at a given distance in space. Still, it could be argued that audiovisual co-localization was mostly inferred. In fact, the realistic biological motion stimuli might have enhanced the association between sound and image as part of the same audiovisual event. Moreover, the instructions given to the participants might have contributed to this association. As Arnold and collaborators [18] demonstrated, instructions can be an important factor in the emergence of compensatory evidence.

 Despite all these limitations, some hypotheses on what is guiding compensation can be further discussed, taking into account our results. First of all, assuming that our stimuli were perceived as co-localized in the “audiovisual depth cues” condition, co-localization of auditory and visual stimuli seems to be an important factor to get compensation for the relatively slow speed of sound. However, co-localization does not seem mandatory for compensation of sound propagation velocity. Alais and Carlile have found evidence of compensation for sound propagation velocity by providing auditory depth cues while keeping the visual stimuli at a fixed distance [5]. In their study the ratio of direct-to-reverberant energy was used as an auditory depth cue, but the visual stimulus was fixed at 57 cm from the observer and primarily used as a reference point in time. Furthermore, their results show that the compensation effect relies on the robustness of the auditory depth cues. Thus, the authors concluded that reliable auditory depth cues together with a task-relevant situation are sufficient in order to activate compensation for the sound propagation velocity. These results shift the focus from co-localization to specific depth cues, when we try to uncover the reason for having compensation in some experimental conditions. Our results agree with the idea that a powerful auditory depth cue is necessary in order to get evidence for sound propagation velocity compensation: only when we add the binaural recordings of sound steps do we get clear evidence for such compensation (see Figure 5). Nevertheless, we cannot state that co-localization is not important, since stimuli were co-localized in this condition. A new experiment including a condition where only the auditory stimuli are informative about distance should be performed in order to give a clearer answer to this question. However, taking into account our results, we can assert that auditory depth cues may play a primary role in the activation of mechanisms of simultaneity constancy. 

 We also want to draw attention to other characteristics of our stimuli and experimental set-up that might contribute to the study of compensation of sound propagation velocity. Different studies showed that evidence of compensation for stimuli distance is difficult to observe when complex stimuli are used [12,18]. Moreover, Arnold and colleagues [18] were unable to find reliable evidence of perceptual compensation even using audiovisual stimuli where causal attributions could be made. However, their experimental set-up suffered from conflicting visual depth cues. Indeed, most of the studies reporting evidence for compensation have used low-level stimuli without causal relations between events and multimodal signals. Therefore, the role of causal attribution in the activation of sound propagation velocity compensation remains a valid and pressing issue. Point-light displays with biological motion can be considered as complex stimuli since they convey dynamic information and involve causal relations between events and multimodal signals. For this reason, they seem to be a powerful tool to test the effect of stimuli complexity over compensation for stimuli distance. We know that some stimuli characteristics like biological motion [6] and conventional orientation [20] can affect the PSS in an audiovisual simultaneity judgment task. These findings highlight the importance of naturalistic representations in the judgment of synchrony. However, we cannot clearly state that, like certain depth cues, causality plays a key role in the activation of perceptual compensatory mechanisms. On the one hand, our results support the existence of compensation for sound propagation velocity; but on the other hand, biological motion and causal relation between stimuli were presented in all conditions, preventing conclusive interpretations on this topic. Nevertheless, we can assert that when causal attributions and conflicting depth cues are presented in the same audiovisual stimulus, evidence for compensation becomes difficult to find. This may explain some previous results [18] and can also account for some parts of our data in the “reduced depth cues condition”, where we presented a representation of the feet taking one step and step sounds that could be causally related to the visual step, but where both auditory and visual depth cues were greatly impaired. 

 Our work was carefully designed taking into account several problems pointed out in previous studies [5,8,9,15]. More specifically, we avoided the use of simple and stationary stimuli with poor distance cues. We also used bimodal stimuli with a causal relation between them, as well as familiar stimuli. By using a PLW and real step sounds, we provided the participant with a type of stimulus that occurs in everyday life. Indeed, the finding of compensation for sound propagation velocity with such stimulus might be evidence that, apart from relying in some depth cues, this mechanism also uses knowledge from previous experience. In real-life situations we are always exposed to a certain audio delay. Despite this delay being highly variable, a long time of exposure to it could lead to some kind of temporal recalibration. This approach is in accordance with the work of Heron and collaborators [22] where, after a brief phase of exposure to the natural sound lag of a distant audiovisual stimulus, participants shifted lag expectations. Moreover, the work of Virsu and collaborators has shown that simultaneity constancy can be learned in natural interactions with the environment and without explicit feedback. Effects of simultaneity constancy appear to be long-lasting and modality specific [23]. Also, studies comparing the perception of synchrony in adults and infants have found that the thresholds for asynchrony detection are modified as we get older [24], further showing that synchrony perception is affected by our history of exposure to certain audio delays.

 In summary, from the present findings from our lab, we should stress that the presence and quality of depth cues appears to be crucial for the activation of a mechanism of compensation for sound propagation velocity. However, we cannot clearly state that participants used an implicit knowledge of both absolute distance and speed of sound in the air. This would indeed be a remarkable computational feat. An alternative explanation is that visual and auditory naturalism, together with perceptual coherence, allowed subjects to recall previously learned audiovisual timings. We claim this to be the reason why evidence for this kind of compensation is stronger in more naturalistic stimulation conditions. New studies are needed to clarify the role of several variables on the simultaneity constancy mechanism, namely instruction, relation between the two modalities, and stimulus familiarity. Studies manipulating these variables will clarify the role of implicit knowledge and recalibration factors. Further research might also explore the implications of such findings to applied fields such as audiovisual systems and immersive environments, where we foresee promising technological applications [25]. 

 In conclusion, our work brings a new contribution for a recurring discussion in audiovisual perception: the existence of a perceptual mechanism that compensates for natural audio lags. Moreover, we show that when a compensatory pattern of response does occur, depth cues are crucial in defining the amount of audio lag compensation. 

## Supporting Information

Video S1 **Representation of the visual stimulus used in the “audiovisual depth cues” condition.**
(WMV)
