# Correction: Depth Cues and Perceived Audiovisual Synchrony of Biological Motion

**DOI:** 10.1371/annotation/d0e27a68-ad6d-452f-bfca-337487fc933c

**Published:** 2014-01-03

**Authors:** Carlos César Silva, Catarina Mendonça, Sandra Mouta, Rosa Silva, José Creissac Campos, Jorge Santos

There was a missing affiliation for the second author, Catarina Mendonça. The correct affiliations for Catarina Mendonça are:

Department of Psychology, School of Medicine and Health Sciences, Carl von Ossietzky University, Oldenburg, Lower Saxony, Germany

Department of Signal Processing and Acoustics, Aalto University, Finland

